# Altered mitochondrial function in fibroblast cell lines derived from disease carriers of spinal muscular atrophy

**DOI:** 10.1038/s43856-024-00515-w

**Published:** 2024-05-15

**Authors:** Rachel James, Kiterie M. E. Faller, Ewout J. N. Groen, Brunhilde Wirth, Thomas H. Gillingwater

**Affiliations:** 1 Edinburgh Medical School: Biomedical Sciences, University of Edinburgh, Edinburgh, UK; 2 Euan MacDonald Centre for Motor Neuron Disease Research, University of Edinburgh, Edinburgh, UK; 3 UMC Utrecht Brain Center, Department of Neurology and Neurosurgery, University Medical Center Utrecht, Utrecht, the Netherlands; 4 Institute of Human Genetics, Center for Molecular Medicine Cologne, and Center for Rare Diseases Cologne, University Hospital of Cologne, University Cologne, 50931 Cologne, Germany; 5 Present Address: IRR Chemistry Hub, Institute for Regeneration and Repair, University of Edinburgh, Edinburgh, UK

**Keywords:** Neurodegeneration, Neuromuscular disease

## Abstract

**Background:**

Spinal muscular atrophy (SMA) is an autosomal recessive childhood-onset neuromuscular disease with a carrier frequency of ~1:50. Mitochondrial abnormalities are widespread in patients with SMA. Disease carriers for SMA (i.e., the parents of patients with SMA) are viewed as asymptomatic for SMA disease. As far as we are aware, mitochondria have not been previously examined in SMA carriers, yet as they are maternally inherited, mitochondrial function in SMA carriers has putative implications for disease pathogenesis.

**Methods:**

Fibroblast cell lines derived from SMA carriers and controls were obtained from two different sources and cultured under standard conditions. The mitochondrial membrane potential, reactive oxygen species (ROS) production, citrate synthase activity, and bioenergetic analysis were examined as measures of mitochondrial function. The mitochondrial genome was also sequenced in a subset of the fibroblast cell lines to identify any mitochondrial DNA variants.

**Results:**

Here, we show a depolarized mitochondrial membrane potential, increased levels of reactive oxygen species, and reduced citrate synthase activity in SMA carriers compared with controls. A likely pathogenic variant in the *MT-CO3* gene (which encodes subunit III of cytochrome c oxidase) was also identified in a paternal carrier.

**Conclusions:**

This study was conducted as a preliminary investigation of mitochondrial function in SMA carriers. Our findings suggest that disease carriers of SMA show differences in mitochondrial function, indicative of a subclinical mitochondrial phenotype. Further investigation in a larger sample set is warranted.

## Plain language summary

Spinal muscular atrophy (SMA) is a disease that mostly affects children in which the muscles become weaker over time, and often leads to death in untreated individuals. It is caused by a defective gene that children often inherit from their parents. The parents of children with SMA are known as disease carriers if they do not show any symptoms of SMA themselves. We studied skin cells from the parents of people with SMA and found changes in a component of the cells called the mitochondria. These changes are not normally present in healthy individuals. Further work is needed to fully understand the implications of our findings for those with SMA and their parents.

## Abstract (web summary)

James et al. compare mitochondrial function in fibroblast cell lines from disease carriers of spinal muscular atrophy (SMA) and control individuals. They report altered mitochondrial activity in SMA carriers, suggesting that carriers have a subclinical mitochondrial phenotype.

## Introduction

Spinal muscular atrophy (SMA) is a predominantly childhood-onset neuromuscular disease caused by loss-of-function mutations in the survival of motor neuron 1 (*SMN1*) gene^1^. *SMN1* mutations show an autosomal recessive inheritance pattern, with a carrier frequency of around 1:50^2^. SMN protein in humans is obtained from two highly similar genes, *SMN1* and *SMN2*. Patients with SMA show reduced levels of SMN protein due to insufficient compensation of protein expression by *SMN2*. Without treatment and in its most severe forms, SMA leads to muscle atrophy, weakness, paralysis, and premature death. Current therapies restore SMN protein levels and have led to improved clinical outcomes for many patients^3^. However, not all patients respond to treatment and there is a need for combinatorial therapies.

To explore the possibility that inherited mitochondria may exhibit a subclinical phenotype, we examined mitochondrial function in fibroblast cells from SMA carriers, specifically, parents of patients with SMA. Mitochondrial abnormalities are found in patients and experimental models of SMA^4^. Moreover, in a mouse model of SMA, changes in mitochondrial morphology at an early postnatal stage^5^ suggest that mitochondrial function may be affected *in utero*. Mitochondria are maternally inherited, with this inherited population dividing to ultimately populate all cell types of the body^6^. Thus, the fitness of this initial population of mitochondria is critical for optimal health during development and beyond. Disease carriers of SMA have altered levels of SMN protein but are asymptomatic. Here, we show changes in the mitochondrial membrane potential, ROS levels, and citrate synthase activity in SMA carriers compared with controls.

## Methods

### Cell culture

Fibroblast cell lines were obtained from the HGRI Sample Repository for Human Genetic Research at the Coriell Institute for Medical Research (Camden, NJ, USA – cell lines GM03651; GM01650; GM03813; GM03814; GM03815 deposited with informed consent), or a collection maintained by Brunhilde Wirth (University of Cologne, Cologne, Germany) that were deposited with informed consent and approval of the ethics committee of the University Hospital of Cologne (approval numbers 04–138 and 13–022). They were not tested for mycoplasma contamination. All cell lines were authenticated regarding *SMN1*/*2* genotype (Supplementary Data). The sex of each individual from whom each line was derived from was known (Supplementary Data). Fibroblasts were cultured in Dulbecco’s modified Eagle’s medium supplemented with 15% foetal bovine serum, 1% non-essential amino acids, and penicillin/streptomycin (all Gibco Thermo Fisher Scientific, Waltham, MA, USA) at 37 °C in a humidified chamber of 95% air and 5% CO_2_.

### Mitochondrial membrane potential

Fibroblasts were plated (0.5 × 10^4^ cells/well) in triplicate into 96-well tissue culture plates and incubated overnight. Cells were incubated in a non-quenching concentration^7^ (25 nM) of tetramethylrhodamine, ethyl ester (TMRE) for 30 min at 37 °C, in accordance with the manufacturer’s guidelines (Abcam, Cambridge, UK). Relative fluorescence units (RFU) were corrected for mitochondrial content by the amount of TOMM20 protein (Invitrogen, Waltham, MA, USA; PA5-52843; 1:1,000 dilution), obtained independently by western blotting.

### Mitochondrial ROS production

Fibroblasts were plated (3.0 × 10^4^ cells/well) in triplicate into 96-well tissue culture plates and incubated overnight. Cells were treated with 5 μM MitoSOX Red for 30 min at 37 °C as described^8^ and in accordance with the manufacturer’s guidelines (Invitrogen). RFU were corrected for mitochondrial content by the amount of TOMM20 protein, obtained independently by western blotting.

### Citrate synthase activity

Citrate synthase activity was measured in 1 × 10^6^ cells in accordance with the manufacturer’s guidelines (Abcam). Citrate synthase activity was corrected by the amount of cellular protein, obtained independently by bicinchoninic acid assay (Thermo Fisher Scientific).

### Bioenergetic analysis

Fibroblasts were plated at experimentally determined densities (3.0–5.0 × 10^4^ cells/well; 3–5 replicates for each cell line) into Seahorse XF 24-Cell Culture Microplates (Agilent Technologies, Santa Clara, CA, USA) and incubated overnight. Bioenergetic analyses were performed using the Seahorse XFe24 Analyzer (Agilent Technologies), in accordance with the manufacturer’s guidelines. The Mito Stress Test protocol was performed with optimized doses of oligomycin (1 μM), FCCP (2 μM), and rotenone/antimycin (both 0.5 μM) to determine oxygen consumption rate (OCR) and extracellular acidification rate (ECAR). OCR and ECAR values were normalized to protein content, measured independently by sulforhodamine B assay^9^. Data were analyzed using Seahorse Analytics software.

### Mitochondrial genome analysis

Genomic DNA was prepared from cell pellets of the SMA family trio in Cohort 1 (obtained from the Coriell Institute for Medical Research). DNA was referred to the NHS Highly Specialised Service for Rare Mitochondrial Disorders (Newcastle, UK) for mitochondrial DNA (mtDNA) analysis. Variants were identified by Next Generation Sequencing of mtDNA (Ion Torrent; Thermo Fisher Scientific) and alignment to the revised Cambridge reference sequence for human mtDNA (NC_12920.1) (Supplementary Data).

### *SMN* genotyping

Genomic DNA was prepared from cell pellets using the QuickGene DNA Tissue extraction kit (FUJIFILM Wako Chemicals Europe GmbH, Neuss, Germany). Multiplex ligation-dependent probe amplification was used to identify *SMN1* and *SMN2* copy number (SALSA MLPA Probemix P021 SMA; MRC Holland, Amsterdam, the Netherlands).

### Statistics and reproducibility

Statistical analyses were performed using GraphPad Prism (version 9.0.0 for Windows; GraphPad Software, San Diego, CA, USA). Normality of the data were determined using the Shapiro–Wilk test. Based upon normality, the data were then analyzed by two-tailed Student’s *t*-test or Mann–Whitney test. ‘Independent’ means that each experiment was performed on distinct weeks and from different cell passages and/or stocks. Experimental ‘*n*’ was used was used for statistical analysis^10^. Technical replicates for each experiment were averaged to generate an experimental mean, which was separately analyzed. Each experiment was performed at least three-times. *P* < 0.05 was considered statistically significant.

### Reporting summary

Further information on research design is available in the Nature Portfolio Reporting Summary linked to this article.

## Results

We used two independent cohorts of fibroblast cell lines to examine mitochondrial function in SMA carriers. All controls were homozygous for the functional allele of *SMN1*, while the carriers were heterozygous. The groups were balanced by age but not sex.

**Fig. 1:**
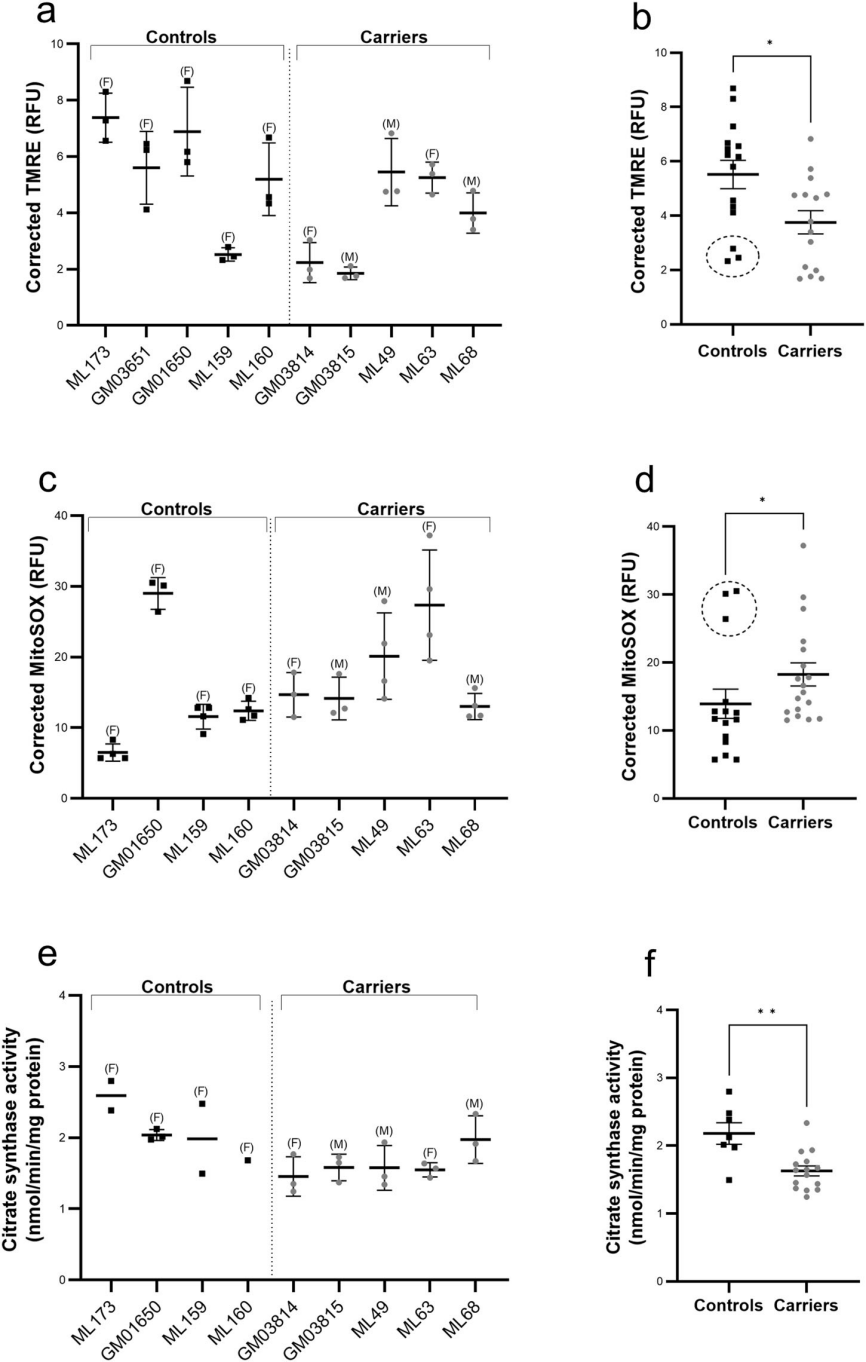
Altered mitochondrial function in fibroblast cells of SMA carriers. Cell-based assays were used to measure the mitochondrial membrane potential (by TMRE) (**a**, **b**) mitochondrial reactive oxygen species (by MitoSOX) (**c**, **d**), and citrate synthase activity (**e**, **f**). Compared with controls, spinal muscular atrophy (SMA) carriers show a reduced mitochondrial membrane potential (Controls: 5.5 ± 0.5 RFU, Carriers: 3.8 ± 0.4 RFU; Student’s *t*-test, *P* = 0.0143), increased ROS levels (Controls: 13.9 ± 2.1 RFU, Carriers: 18.2 ± 1.7 RFU; Mann–Whitney test, *P* = 0.0266), and reduced citrate synthase activity (Controls: 2.2 ± 0.2 RFU, Carriers: 1.6 ± 0.1 nmol/min/mg protein; Student’s *t*-test, *P* = 0.0016). In (**a**) and (**c**), *n* = 3 or 4 independent experiments (3 technical replicates/experiment). In (**e**) mostly *n* = 3 independent experiments; but due to growth issues, certain control lines were assayed < 3-times (ML159/ML173, *n* = 2; ML160, *n* = 1). Mean ± SD are shown. The sex of the individual from which the fibroblasts were obtained is indicated in brackets. F, female; M, male. In (**b**), (**d**), and (**e**), each ‘*n*’ was analysed as an experimental replicate. ML160 was excluded from analysis in (**e**) due to lack of replication. ^*^P < 0.05, Controls vs. Carriers. Mean ± SEM are shown. Specific control cell lines (ML159 in A; and GM01650 in B; shown in dashed circle) may be outliers for the assay but were not excluded from analyses. RFU, relative fluorescence units. TMRE, tetramethylrhodamine, ethyl ester.

The mitochondrial membrane potential indicates the polarized state and functional status of mitochondria. We examined the membrane potential using TMRE, a dye that sequesters in mitochondria based upon their membrane potential. Compared with controls, SMA carriers showed a reduced (or more depolarized) mitochondrial membrane potential (Fig. 1a, b). Depolarization of the mitochondrial membrane potential can be caused by altered production of mitochondrial ROS. Therefore, we examined levels of mitochondrial ROS using MitoSOX, a dye that binds to superoxide species in mitochondria. We found an overall increase in ROS production in SMA carriers compared with controls (Fig. 1c, d).

**Fig. 2:**
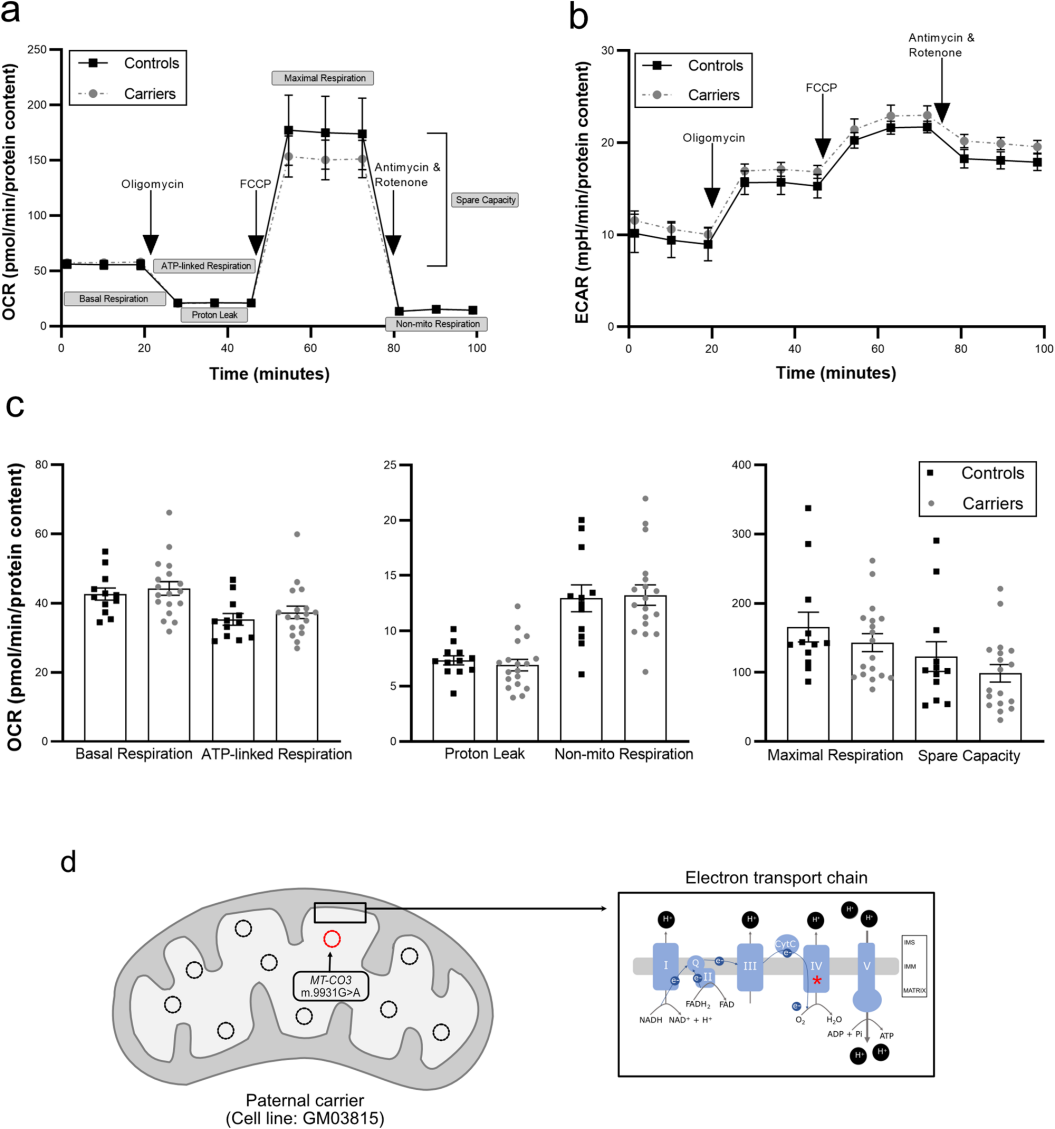
Respiratory bioenergetics and mitochondrial genome analyses in fibroblast cells of SMA carriers. The Seahorse bioanalyzer was used to measure oxygen consumption rate (OCR) (**a**) and extracellular acidification rate (ECAR) (**b**). The Mito Stress Test was performed with drugs that target activity of specific complexes of the electron transport chain: oligomycin (Complex V), FCCP (Complex IV), antimycin (Complex I), and rotenone (Complex III). Specific respiratory parameters were then calculated (indicated for the OCR graph in grey shaded boxes). ECAR reflects medium acidification due to metabolic processes. Kinetic curves (**a**, **b**) are shown for OCR and ECAR in carriers compared with controls. Summary data for OCR are also shown (**c**). No significant differences were detected in spinal muscular atrophy (SMA) carriers compared with controls for any OCR/ECAR parameter (*P* > 0.05, Student’s *t*-test; exact *P*-values are provided in the Supplementary Data). Each cell line was assayed in 2–4 independent experiments, with 3–8 technical replicates per experiment. Failed reactions (low or no respiratory profile) were excluded from further analyses. Combined data are shown in the kinetic curves (**a**, **b**), with each experimental replicate shown in the summary data (**c**). Mean ± SEM are shown. The entire mitochondrial genome was amplified by long range PCR and next generation sequencing in the carrier (GM03814 and GM03815) and patient (GM03813) cell lines from Cohort 1. A likely pathogenic variant (m.9931 G > A p.[Trp242*]) was identified in the *MT-CO3* gene of the paternal cell line (**d**). The mutation was detected at 10% heteroplasmy (indicated by red mtDNA molecule in matrix), meaning that 90% of mtDNA (indicated by black mtDNA molecules in matrix) does not contain this variant. The *MT-CO3* gene encodes subunit III of cytochrome c oxidase (Complex IV) of the electron transport chain (indicated by red asterisk in the boxed image of the mitochondrial membrane region). mtDNA, mitochondrial DNA; IMM, inner mitochondrial membrane; IMS, intermembrane space.

As additional measures of mitochondrial function, we examined bioenergetic activity of the fibroblasts. Citrate synthase is the first enzyme in the tricarboxylic acid cycle and the final pathway for oxidation of nutrients. We observed reduced citrate synthase activity in SMA carrier fibroblasts compared with controls (Fig. 1e, f). However, further analysis of respiratory bioenergetics found no significant changes in measures of OCR (reflecting oxidative phosphorylation) or ECAR (medium acidification broadly due to metabolic processes) (Fig. 2a–c). Taken together, these have revealed modest changes in mitochondrial function in SMA carriers compared with controls.

Finally, to determine whether mtDNA variants are associated with SMA carriers and/or patients, the mitochondrial genome was sequenced in carrier (maternal, GM03814 and paternal, GM03815) and patient (GM03813) cell lines from Cohort 1. This identified a likely pathogenic variant (m.9931 G > A p.(Trp242*])) at ~10% heteroplasmy in the *MT-CO3* gene of the paternal cell line (Fig. 2d). The *MT-CO3* gene encodes subunit III of cytochrome *c* oxidase (i.e., Complex IV), but as the variant is only present in 10% of mtDNA molecules, its functional impact is unclear.

## Discussion

The aim of this study was to determine whether fibroblast cell lines from asymptomatic disease carriers of SMA show altered mitochondrial function. Accordingly, we identified changes in the mitochondrial membrane potential, ROS levels, and citrate synthase activity in carriers compared with controls. The phenotype identified may reflect altered baseline mitochondrial activity in the fibroblasts. A depolarized mitochondrial membrane potential and increased ROS would typically be associated with a less healthy population of mitochondria. However, as mitochondrial stress can be viewed as means to build cellular resilience^11^, at least in certain cell types^12^, a more stressed population of mitochondria may not necessarily be harmful. Speculatively, depolarized mitochondria should be targeted for degradation through ubiquitin pathways^13^. SMN is proposed to play a role in ubiquitination^14^, therefore it could be interesting to investigate this process further in carriers. Alternatively, the phenotype may reflect a response to the artificial environment imposed by cell culture, not least the use of antibiotics to examine the function of an organelle of bacterial ancestry^15^. In this case, the phenotype may suggest that carriers have a less robust response to cellular stress.

The mtDNA variant identified in a paternal carrier has only been reported once previously in a case of mitochondrial myopathy with recurrent myoglobinuria^16^. It was detected at considerably lower heteroplasmy (10%) than the 50–60% heteroplasmy associated with primary mitochondrial diseases^17^. Being found in a paternal carrier, it will not be transmitted to the patient. Moreover, the absence of mutations in the maternal carrier and/or patient lines does not exclude their presence as mtDNA can be lost during culture of primary fibroblasts^18^. No marked differences in respiratory phenotype described by OCR/ECAR parameters were identified under the conditions examined. One consideration here is the use of a high glucose medium for cell culture: a bioenergetic mitochondrial phenotype may not be observed unless cultured cells are grown in a medium that forces them to rely on oxidative phosphorylation^19^.

This study was conducted as a preliminary investigation of mitochondrial function in SMA carriers. Altogether, it can be concluded that in this sample set of fibroblasts, SMA carriers show differences in mitochondrial function. However, a limitation of our study is the small sample size, impacted by limited availability of cell lines from carriers as well as matched controls. Future experiments would benefit from a more homogenous sample set (in terms of e.g., age, *SMN2* copy number, SMA clinical subtype). Indeed, inspection of the data shows an apparent influence of age in certain assays (e.g., the TMRE assay; Fig. 1a), with age having a well-described influence on mitochondrial function (see e.g^20^.). Although direct extrapolation of these findings to other cell types and/or the in vivo situation is not possible, given the increasingly recognized role of mitochondria in cell fate processes^21^, including those related to neurodevelopment^22^, further understanding of the possible inheritance of altered mitochondrial function in patients may benefit families with SMA and possibly even other diseases with mitochondrial dysfunction as a pathological hallmark. Moreover, the identification of cellular phenotypes in SMA carriers suggests that more extensive investigations of subclinical phenotypes are now warranted as it could help better decipher the complexity of the disease, and ultimately lead to the discovery of combinatorial therapy targets.

### Supplementary information

Supplementary Data

Description of Supplementary Data

Reporting Summary

## Data Availability

The data supporting Figs.1 and 2 are provided in the Supplementary Data. All raw data are available from the corresponding author upon request.
