# Supplementary material for: Altered mitochondrial function in fibroblast cell lines derived from disease carriers of spinal muscular atrophy
Source: Commun Med (Lond). 2024 May 15;4:86. doi: 10.1038/s43856-024-00515-w (PMC11096342; doi:10.1038/s43856-024-00515-w)
Supplement: Supplementary file 2 — Description of Supplementary Data [file 43856_2024_515_MOESM2_ESM.docx]

**Description of Additional Supplementary Source Data File**

**File Name:** Source Dataset

**Description:** Demographic characteristics of all fibroblast cell lines used in the study (Fibroblast cell lines). Data from cell-based assays of mitochondrial function, as indicated on the individual worksheets: TMRE assay, Fig. 1a, b; MitoSOX assay, Fig. 1c, d; Citrate synthase activity, Fig. 1e, f; Seahorse bioenergetic assay, Fig. 2a, b, c. These data were used to generate all the graphs in Figs. 1 and 2, and obtain *P*-values by statistical testing.
